# Interventions for Improving Psychosocial Adjustment in Nursing Home Residents: A Network Meta-Analysis

**DOI:** 10.1093/geroni/igaa057.894

**Published:** 2020-12-16

**Authors:** Yanyan Zhang, Huimin Xiao, Binbin Yong

**Affiliations:** 1 Fujian Medical University, Fuzhou, Fujian, China; 2 Fujian Medical University, Fuzhou, China

## Abstract

This study aimed to identify the evidence of interventions for improving psychosocial adjustment in older adults relocating to nursing homes. We followed PRISMA-NMA guidelines to conduct a network meta-analysis. 12 electric databases were used to search for eligible randomized controlled trials (RCTs) and clinical trials (CCTs) from inception to August 27th, 2019. Two reviewers independently conducted article screening, data extraction, and risk of bias appraisal with the Revised Cochrane risk-of-bias tool for RCT and Risk Of Bias for CCT. The network plots were plotted to provide a visual representation of the evidence base. Bayesian fixed-effects pairwise and network meta-analysis were exhibited in the forest plot. A Bayesian network meta-analysis was performed on 30 eligible RCTs with 2119 participants, and 12 eligible CCTs with 491 participants. The quality of the most included studies was rated as moderate in RCTs and low in CCTs. Treatment effects showed that compared to conventional treatment, group reminiscence and group counseling resulted in significant improvements in loneliness (MD: 11, 95%, CI: 2.7–17, SUCRA: 99.5%; MD: 7.7, 95%, CI: 0.53–15, SUCRA: 53.9%, respectively). Similar results were obtained for art therapy (MD: 5.5, 95%CI: 0.8–10, SUCRA: 97.3%) in self-esteem. The model fit was good, and the inconsistency was low. Group reminiscence, group counseling, and art therapy are recommended for reducing loneliness and enhance self-esteem in nursing practice.

